# Comitant strabismus etiology: extraocular muscle integrity and central nervous system involvement—a narrative review

**DOI:** 10.1007/s00417-022-05935-9

**Published:** 2023-01-21

**Authors:** Bernat Sunyer-Grau, Lluïsa Quevedo, Manuel Rodríguez-Vallejo, Marc Argilés

**Affiliations:** 1 School of Optics and Optometry, Universitat Politècnica de Catalunya, Terrassa, Spain; 2 Department of Ophthalmology of VITHAS Almería Hospital, Qvision, 04120 Almería, Spain

**Keywords:** Strabismus, Etiology, Central nervous system, Extraocular muscles

## Abstract

Strabismus is not a condition in itself but the consequence of an underlying problem. Eye misalignment can be caused by disease, injury, and/or abnormalities in any of the structures and processes involved in visual perception and oculomotor control, from the extraocular muscles and their innervations to the oculomotor and visual processing areas in the brain. A small percentage of all strabismus cases are the consequence of well-described genetic syndromes, acquired insult, or disease affecting the extraocular muscles (EOMs) or their innervations. We will refer to them as strabismus of peripheral origin since their etiology lies in the peripheral nervous system. However, in most strabismus cases, that is comitant, non-restrictive, non-paralytic strabismus, the EOMs and their innervations function properly. These cases are not related to specific syndromes and their precise causes remain poorly understood. They are generally believed to be caused by deficits in the central neural pathways involved in visual perception and oculomotor control. Therefore, we will refer to them as central strabismus. The goal of this narrative review is to discuss the possible causes behind this particular type of eye misalignment and to raise awareness among eyecare professionals about the important role the central nervous system plays in strabismus etiology, and the subsequent implications regarding its treatment. A non-systematic search was conducted using PubMed, Medline, Cochrane, and Google Scholar databases with the keywords “origins,” “causes,” and “etiology” combined with “strabismus.” A snowball approach was also used to find relevant references. In the following article, we will first describe EOM integrity in central strabismus; next, we will address numerous reasons that support the idea of central nervous system (CNS) involvement in the origin of the deviation, followed by listing several possible central causes of the ocular misalignment. Finally, we will discuss the implications CNS etiology has on strabismus treatment.


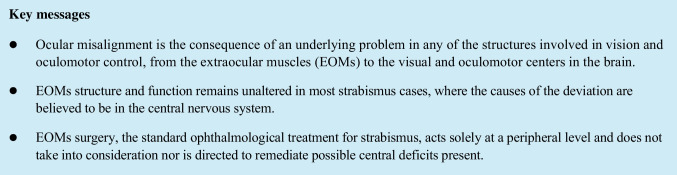


## Introduction

**Fig. 1:**
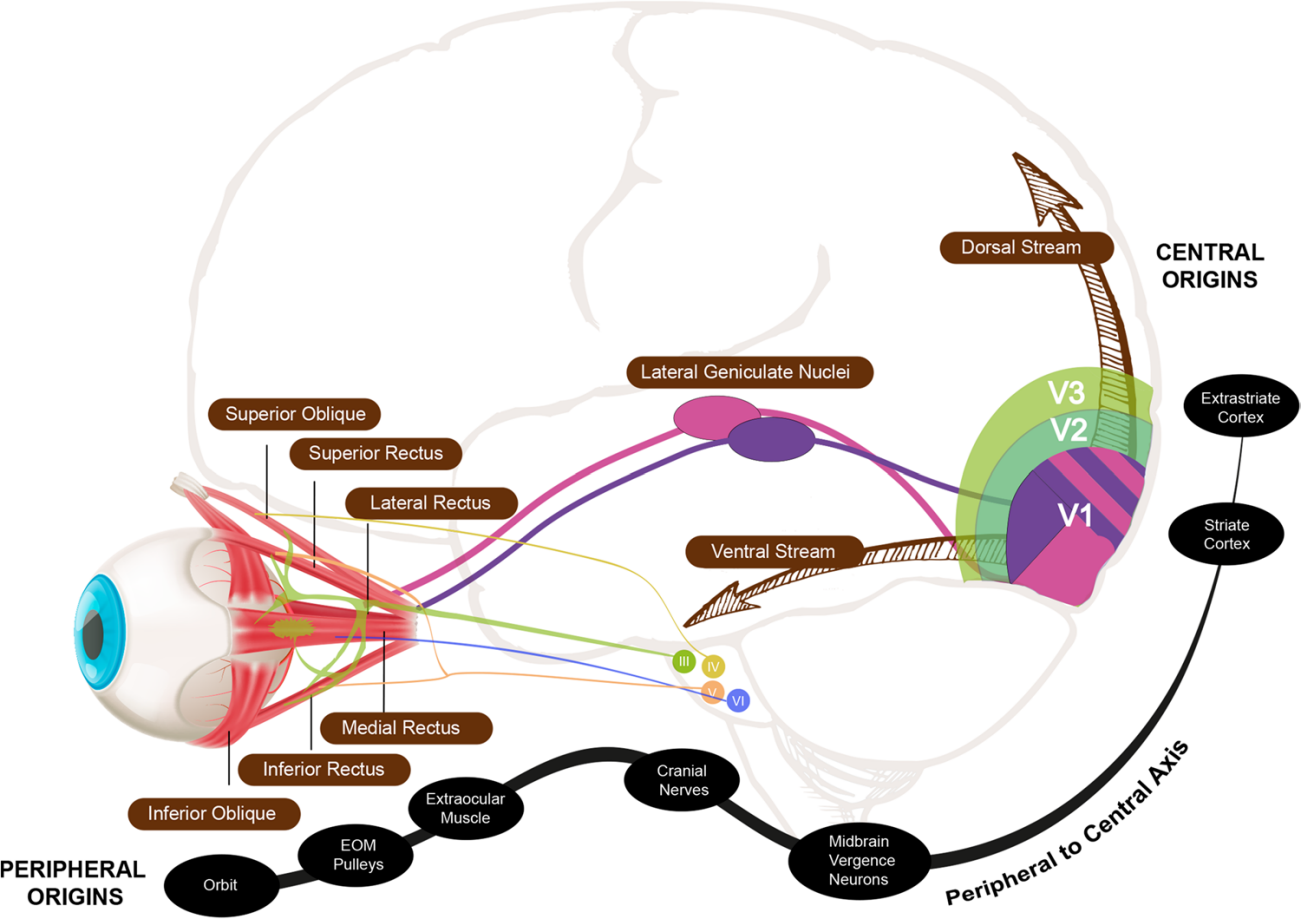
Peripheral-central axis. It comprises structures of the visual and oculomotor systems, from the eye (periphery) to the brain (center). It includes the orbit, the extraocular muscles (EOMs), their pulleys, the nerves and their nuclei, the midbrain fusion centers, the lateral geniculate nucleus (LGN), and the primary (V1) and secondary visual areas

Strabismus is a common disorder that affects from 3 to 6% of the worldwide population [1–4]. The American Academy of Ophthalmology (AAO) defines strabismus as a misalignment of the eyes that may be congenital or acquired [5]. The direction, magnitude, and frequency of the deviation can vary widely between patients. The characteristics of the deviation can also change depending on the position of gaze (incomitance), and the viewing distance, near versus far. In addition, double vision, suppression, or anomalous sensory correspondence might be present, and in 12.5% of cases, the eye turn and amblyopia occur concurrently [6]. Strabismus can present itself in multiple forms, what some authors have called “strabismus polymorphy” [7]. This suggests that the eye turn is a consequence of a vast number of conditions of multiple possible origins, influenced by either genetic or environmental factors, or a combination of both [8–10]. Strabismus is not a single entity nor a condition in itself. The eye misalignment is a manifestation/sign of an underlying problem involving one or multiple components of the visual and oculomotor systems along the peripheral-central axis (Fig. 1), from the extraocular muscles (EOMs) themselves, their pulleys [11] and their innervations, to all brain areas involved in visual perception and oculomotor control: midbrain fusion centres [12], the lateral geniculate nucleus (LGN) and striate and extrastriate areas [13].

Strabismus of peripheral origin account for 5–25% of all strabismus cases [1, 4, 7, 14] and are the consequence of either complex well-described genetic syndromes [12, 13] or due to acquired insult to the eye or its innervations. Peripheral strabismus are predominantly incomitant and can be caused by ocular or orbital trauma, craniofacial abnormalities, connective tissue disorders and syndromes, muscular dystrophies, genetic myopathies, and cranial nerve syndromes and palsies [15–18].

On the other hand, the majority of strabismus, 75–95% of all strabismus cases [1, 4, 7, 14], are concomitant, non-restrictive, non-paralytic, or developmental. They are not related to specific syndromes, and their genetic associations remain poorly understood [7, 13, 19–29]. They are generally believed to be caused by deficits in the central neural pathways involved in visual perception and oculomotor control [7, 30–36]. Although several possible mechanisms have been hypothesized, the specific central origins of strabismus remain elusive.

The aim of this study was to review and discuss the latest evidence about the etiology of the most common, and at the same time, the less known, types of ocular deviations: non-restrictive, non-paralytic, and developmental strabismus. For this purpose, the EOM integrity in these cases is described first, addressing the following question “Are there any abnormalities in the EOMs in central strabismus?” Then, numerous reasons that support the idea of central involvement in the origin of the deviation are addressed, and finally, several possible central causes of the ocular misalignment are listed. With this review, we aim to create awareness among eyecare professionals about the important role the central nervous system plays in strabismus etiology, and the subsequent implications regarding its treatment.

The scientific literature on the possible causes of strabismus, in particular regarding the integrity of the EOMs and the structure and function of the CNS, was searched. We used PubMed, Medline, Cochrane, and Google Scholar databases. We did not use restrictions for date nor language of publication; databases were last searched on June 2022. The keywords “origins,” “causes,” and “etiology” were combined with “strabismus.” A snowball approach was also used to track down relevant references.

## Extraocular muscle integrity in concomitant, non-paralytic, strabismus

Despite minor differences, gross anatomical, ultra-structural [37], and histological [38] organization of EOMs in concomitant developmental strabismus are similar to that of EOMs without strabismus. High-resolution, surface coil magnetic resonance imaging (MRI) showed normal horizontal rectus muscle path lengths in strabismic patients compared to normal controls [39]. Sizes, paths, structure, and innervation of horizontal rectus EOMs and in connective tissues in the pulley system revealed no significant differences between orthotropic and naturally or artificially strabismic monkeys under histological examination and MRI [40]. On the other hand, high-resolution MRI showed medial rectus muscle to be 39% larger in esotropic patients than in controls [41]. Surprisingly, in the same individuals, lateral rectus muscle cross-sections in esotropia were up to 28% larger but only significantly larger in one plane [41]. Hao et al. 2016 reported similar results: rectus pulleys were found to be displaced differently in subjects with A, V, and Y, pattern exotropia but normal in concomitant exotropia. On the other hand, medial rectus muscle size was found to be significantly reduced in concomitant exotropia compared to normal subjects and individuals with pattern exotropia (*P* < 0.05) [42]. Other authors reported extensive histological and microscopic abnormalities in strabismic EOMs such as disorganization and atrophy of skeletal muscle fibers, vacuolation and degeneration of myofibrils, accumulation of lipid droplets, and clustering of mitochondria and autophagic processes [43]. Interestingly, all the above-mentioned defects were also seen in the EOMs of a strabismic patient with Down syndrome, a condition both associated with eye misalignment and central nervous system (CNS) defects.

Abnormalities at molecular and gene expression levels in the EOMs appear to be more prominent. Intermediate filament protein distributions were found to be different in EOMs of patients with concomitant exotropia compared to normal subjects [44]. Altick et al. (2012), observed differences in gene expression between strabismic and normal human EOMs in genes associated with signaling, calcium handling, mitochondria function and biogenesis, and energy homeostasis [35]. Moreover, Altick found a decrease in the expression of contractility genes and an increase of extracellular matrix-associated genes. Similar findings of altered gene expression have been reported by other authors. Zhu et al. (2013) found reduced expression of seven myogenesis-related genes in EOMs of patients with concomitant strabismus [45], and Agarwal et al. (2016) reported downregulation of muscle proteins and upregulation of expression of collagens, regulators of collagen synthesis and degradation, connective tissue growth factor (CTGF), and growth factors controlling extracellular matrix (ECM) [46].

These studies suggest that some degree of peripheral muscular abnormality is present in concomitant strabismus. However, it remains unresolved whether the presence of anatomical, histological, and molecular abnormalities in EOM structure are the cause of the ocular deviation, or rather a consequence of altered function, as seen in other muscles [34, 35, 43, 44], for example, in stroke victims where the loss of function leads to abnormalities in skeletal muscle mass and anatomy [47, 48], decrease in fiber length, and change in pennation angle [49]. Mechanical and microstructural changes of skeletal muscle have been reported after changes in function [50], immobilization of skeletal muscles with or without stroke led to atrophy, and deterioration of the mechanical properties of the muscle.

Skeletal muscles, and therefore extraocular muscles [51], have a high degree of adaptability. Muscle fibers contain myofibrils, which are essentially long chains of sarcomeres, the contractile units of skeletal muscle. Skeletal muscles adapt their lengths by addition or subtraction of sarcomeres at the extremes of myofibrils to maintain optimal function. Length adaptation of extraocular muscles can occur in response to muscle position [51], muscle stimulation [52–54], and to facilitate binocular alignment [51, 55]. The cause of abnormal muscle length paths in strabismus could lie well beyond the muscles themselves.

## Strabismus of central origin

The idea that concomitant, non-paralytic, developmental strabismus are caused by deficits in the central nervous system is supported by different arguments. The minor abnormalities found in the EOMs (periphery) in these types of strabismus might be a consequence of altered function rather than the cause of the deviation.

First of all, in most strabismus cases, the subject has no limitation of gaze. Patients with esotropia and exotropia can abduct and adduct either eye effortlessly. Moreover, exotropia can be greater at distance than at near [56], known as divergence excess, where there is no muscular impediment to converge. Likewise, in convergence excess, esotropia is greater at near than at distance, the subject being fully capable to diverge [57].

Strabismus is associated with neurodevelopmental and neurologic disorders such as cerebral palsy [58–60], Down’s syndrome [61–63], neurodevelopment delay [61, 63, 64], intellectual disability [65], and white matter damage of immaturity [66]. Greenberg investigated the prevalence and types of esotropia in a Western population and found that 11.4% of esotropias were associated with CNS defects: cerebral palsy, developmental delay, Down syndrome, and seizure disorder [4]. Likewise, approximately 15% of children with exotropia have associated neurologic abnormalities, cerebral palsy, and developmental delay [67]. Acquired brain injury (ABI), which is any type of brain injury occurring after birth, often leads to eye movement and binocular coordination disorders, including strabismus [68–71]. ABI are predominantly caused by stroke, brain tumor, infection, cerebral hypoxia, or after impact or sudden shake to the head. Lesions can occur at the level of the cranial nerves innervating the EOMs (periphery) or in the brainstem and brain areas involved in oculomotor control (center) [72]. The frequency of ocular misalignment in the presence of brain damage is difficult to report since it depends on the nature, extension, and location of the injury. Fowler found that strabismus was present in 28% of patients with stroke, many of them with no obvious signs of brainstem abnormalities [68]. The higher-than-average prevalence of strabismus in the presence of CNS defects indicates that in many cases, the cause of strabismus lies in the brain. Moreover, these studies depict the relationship between strabismus and obvious, well-defined neurological alterations. More subtle, covert neurological deficits could be the cause of strabismus in a much larger proportion of cases.

Smoking during pregnancy increases the risk of children developing strabismus compared to the children of mothers that did not smoke [73–75]. Fetal development of the oculomotor and visual systems could be particularly sensitive to toxic exposure or smoking-induced fetal hypoxia during the second half of pregnancy. Alterations caused by smoking are more likely to be at the level of the CNS than in the EOMs or their innervations, given its associations to thinning of the cerebral cortex [76], and reduced gray matter volumes and densities in specific areas [77]. Exposure to other potentially neurotoxic substances during pregnancy such as alcohol [78, 79] and drugs [80] is also associated with an increased risk of strabismus.

The concept of strabismic control is another important point indicating central origin and central relevance in strabismus. It is widely recognized that strabismus control plays a significant role in the outcomes of EOM surgery, especially in intermittent exotropia [81, 82]**.** People, whether with strabismus or without, have a certain amount of sensorimotor knowledge related to their eyes and their position in space. In order to grade the amount of control patients have over their deviations, multiple control scores (Table 1) have been developed by different authors [83–86]. They all essentially evaluate strabismic control based on the frequency of the tropic and phoric phase and the quality and speed of the refixation movement after the occlusion of one eye. In addition, some people with strabismus are capable of altering the magnitude of the eye turn, they can do “something” to decrease the deviation, and “something” to increase it even though they are often unable to describe how they do it. In line with strabismus control, in many cases, the deviation worsens when the person is sick, tired, distracted, or absorbed in a highly attention-demanding task. All these unequivocally point to cerebral involvement in eye misalignment pathogenesis [7].

**Table 1:** Newcastle control score. Adapted from Haggerty et al., (2004)[83]

Home control score	0	Squint/monocular eye closure never noticed
	1	Squint/monocular eye closure seen occasionally (< 50% of time child observed) for distance
	2	Squint/monocular eye closure seen occasionally (> 50% of time child observed) for distance
	3	Squint/monocular eye closure seen for distance and near fixation
Clinic control near	0	Manifest only after cover test and resumes fusion without need for blink or refixation
	1	Blink or refixate to control after CT
	2	Manifest spontaneously or with any form of fusion disruption without recovery
Clinic control distance	0	Manifest only after cover test and resumes fusion without need for blink or refixation
	1	Blink or refixate to control after CT
	2	Manifest spontaneously or with any form of fusion disruption without recovery

NCS total = Home + Clinic near + Clinic distance

Strabismus can resolve spontaneously. Infantile esotropia measuring up to 40 PD has been reported to resolve in 46/170 (27%) of patients in the first months of life without treatment [87]. On the other hand, eye misalignment can also worsen over time. Intermittent and variable deviations tend to become constant. Even after successful surgical alignment (less than 10PD from orthotropia), the ocular deviation can reappear. Exotropic drift after an initially successful surgery is a common phenomenon [88–90]. This is another indicator that the cause of the deviation remains untreated and is not related to EOMs structure.

Last but not least, cortical activity changes have been seen after successful treatment in convergence insufficiency, a non-strabismic binocular dysfunction characterized by exodeviation, asthenopia, and in some cases double vision at near [91, 92]. Treatment with vision therapy led to improvement in near point of convergence (NPC), greater positive fusional vergence, reduction in symptoms, and lower near dissociated phoria values. More importantly, improvement in clinical parameters correlated with an increase in functional activity in the frontal areas of the brain, the cerebellum and the brainstem [91, 92].

## Central strabismus etiology

Central strabismus can be the consequence of anatomical and/or functional abnormalities found in any of the brain areas and pathways involved in vision and oculomotor control, including the oculomotor and proprioceptive nuclei in the brainstem, the medial reticular formation, the pontine reticular formation, the superior colliculus, the thalamus, the cerebellum, the corpus callosum, and the occipital lobe, and the extraestriate areas involved in visual processing in the parietal and frontal lobes, the parietal eye field, and the frontal and supplementary eye fields [7, 68–71]. Brain abnormalities can either be the consequence of obvious, identifiable lesions such as the ones caused by trauma, stroke, infection, brain tumor, etc., or the consequence of more subtle insult to the brain, undetectable to testing. In the following section, we will provide with examples of brain abnormalities that could be the cause of strabismus. We will focus on studies reporting reduced gray matter volumes, abnormal brain activity, and abnormal connectivity within and between brain regions in subjects with strabismus. By no means, we believe these examples constitute all the possible brain abnormalities that can result in strabismus; we simply intend to illustrate the variety of defects and locations involved in strabismus pathogenesis.

### Reduced gray matter volumes and abnormal brain activity

Subjects with strabismus present abnormal brain activity in multiple areas, within the primary and secondary visual cortex, but also beyond the occipital lobe. Ouyang et al. (2017) reported reduced gray matter volumes in strabismic patients in the left cuneus [93]. In the lingual gyrus, corresponding to Brodmann area 19 and site to the secondary visual cortex (V2), higher synchrony of spontaneous neuronal activity was observed in amblyopic-strabismic adults [94] and in concomitant strabismus [95] than in normal controls. Moreover, brain activity in the lingual gyrus has been reported to be higher than average in children with infantile esotropia [96]. Shao et al. (2019) reported higher spontaneous brain activity in right and left middle occipital gyrus in amblyopic-strabismic adults [94]. The middle occipital gyrus also corresponds to the secondary visual cortex and is part of the dorsal visual stream. Along the dorsal stream, Chan et al. (2004) found reduced gray matter volumes in the occipital and parietal lobe in strabismic compared to healthy controls [97]. The dorsal stream processes visual information relevant to the position of objects in space and the visual guidance of action [98].

Yang et al. (2014) found increased brain activity in the bilateral precuneus of subjects with Infantile Esotropia compared to healthy controls [96]. Similar findings have been reported by other authors, who have found increased synchrony of spontaneous neuronal activity in the right precuneus [94]. This structure is located on the medial surface of the superior parietal lobe, anterior to the parietooccipital sulcus. It is associated with a variety of functions including cognition, memory, and emotion. In the visual domain, it is involved in visuo-spatial representations, attention, and in the execution, planning, and imagination of movements [99–101].

In the frontal lobe, Shao et al. (2019) found higher synchrony of spontaneous neuronal activity in the precental gyrus (premotor cortex) of both hemispheres and a reduction in the left inferior frontal gyrus [94]. The premotor cortex plays a role in the control of movement, including eye movements. Ouyang et al. (2017) found the right premotor cortex to have reduced gray matter volumes in strabismic patients [93]. In contrast, Chan et al. (2004) observed greater gray matter volumes in the frontal and supplementary eye fields, in the prefrontal cortex, and in the thalamus and basal ganglia in strabismic adults compared to normal controls [97].

Ouyang et al. (2017) also found lower than normal gray matter volumes in strabismic patients in the left middle temporal pole, the left cerebellum posterior lobe, and the right posterior cingulate cortex [93]. Higher synchrony of spontaneous neuronal activity has also been found in the fusiform gyrus and the cerebellum in concomitant strabismus [95].

On the oculomotor and vestibular systems, abnormal neural activity can also result in strabismus. Altered function anywhere in the proprioceptive extraocular circuitry, especially early during development, can derive in loss of feedback control of eye position and hence loss of binocular vision [102–104]. Eye misalignment can also be a consequence of abnormal activity along the vestibular system and pathways [105, 106].

### Abnormal connectivity

#### Cortico-cortical

Numerous cortico-cortical connections suffer alterations in strabismus. Either connections within a given area, such as in V1 in cats [107] or in pathways connecting distant areas. Yan et al. (2010) found white matter volumes to be reduced along the dorsal visual pathway in adults with concomitant exotropia [108]. Huang, Li, Zhang, et al. (2016) reported increased fractional anisotropy (FA) values in the precuneus and medial frontal gyrus of both hemispheres in patients with concomitant strabismus, suggesting enhanced fiber density, axonal diameter, and myelination [109]. In contrast, the authors found decreased FA values in the left superior temporal gyrus. FA is a marker for white matter microstructural state. Decreased FA values are associated with white matter defects. Ouyang et al. (2017) found significantly reduced white matter volumes in the bilateral middle temporal gyrus, the right precuneus and right premotor cortex in concomitant strabismus patients compared to healthy controls [93]. Zhu et al. (2018) observed abnormal functional connectivity in concomitant exotropia patients between the left primary visual cortex (BA17-V1) and the left lingual gyrus/cerebellum posterior lobe, the right middle occipital gyrus, the left precentral gyrus/postcentral gyrus, and the right inferior parietal lobule/postcentral gyrus; abnormal functional connectivity was also found between the right primary visual cortex and right middle occipital gyrus [110].

#### Callosal

The corpus callosum plays a role in the development of human binocularity [111, 112]. Ten Tusscher et al. (2018) found abnormal interhemispheric fibers in the corpus callosum connecting right and left primary visual cortical areas in individuals with infantile esotropia (IE) compared to normal controls [113]. Subjects with IE had a higher amount of these fibers, and their hemispheric distribution was asymmetric, with callosal fibers starting from one primary visual cortex being different from the ones arising from the contralateral homologous areas [113]. Abnormal connectivity of the corpus callosum in the primary and secondary visual cortex has been reported in cats with surgically induced strabismus and in Siamese cats with natural esotropia [114]. The latter suggests that abnormal corpus callosum connectivity is not only a consequence of surgically-induced strabismus but a potential factor in strabismus pathogenesis. This is also supported by studies in cats in which the corpus callosum had been sectioned early on life and consequently displayed strabismus [115–117]. In a study of 13 children with corpus callosum agenesis, strabismus was present in 6 (46%) of the children [118]. Corpus callosum maturation is dependent on visual experience: monocular deprivation [119], complete darkness [120, 121], and strabismus [114, 122] result in a reduction of callosal projections, changes in their distribution and detrimental effects of callosal neuron properties. Moreover, spontaneous neural activity even before eye-opening (in absence of visual input) contributes to normal corpus callosum development [123–125].

#### Dorsal–ventral

It has been hypothesized that a division between the ventral and dorsal streams, as seen in children with Williams syndrome, leads to visual problems and severe visuospatial difficulties. Strabismus is found in a greater proportion of cases of Williams syndrome compared to the normal population [126].

The dorsal stream, involved in processing visuospatial information and planification of visuomotor action [98], has been reported to be particularly compromised in developmental disorders such as Williams syndrome, autism, dyslexia, and in premature infants [126–130]. Atkinson hypothesized that the dorsal stream has specific vulnerability during development [131]. Insult early in development could result in abnormal space representation, altered eye movements, and deficits in visual-directed behaviors such as locomotion, reaching, and grasping, which could be associated to strabismus. Gopal et al. (2020) developed a treatment exercise for amblyopia and strabismus with emphasis on the dorsal stream, both regarding saccadic eye movements and visually guided action such as pointing, and also by improving attention. In their pilot study with 35 subjects with amblyopia, 22 of whom had strabismic amblyopia, stereopsis improved in 34 (97%), and ortophoria was achieved in 15/22 (68%) after 10 one hour sessions [132].

#### In the retino-geniculo-cortical pathway

Abnormal predominance of crossed retinal ganglion cells at the optic chiasm is sometimes associated with strabismus in humans [36, 133]. An unnaturally high number of crossed RGCs is also found in Siamese cats [134] and in albino rats, rabbits, monkeys [135], and also in humans with albinism [136–139]. All previous cases manifest higher than average percentages of strabismus.

## Implications regarding strabismus treatment

The ocular deviation is not a disease in itself but a consequence of an underlying problem ranging from systemic, ocular, and neurological diseases to genetic syndromes and to acquired injury to the structures involved in vision. Lack of understanding of strabismus etiology may prevent treatment from being targeted to the specific causes of the eye misalignment. Optimal medical treatment should be directed to the source. Classical and contemporary standard treatment for strabismus, i.e., EOMs surgery, acts exclusively at a peripheral level. EOM surgery consists of mechanically weakening or strengthening the muscles to correct the eye misalignment. However, in most strabismus cases, there is little or no evidence of abnormalities in the EOMs. In addition, the presence of co-existing CNS abnormalities suggests that the CNS plays a role in strabismus pathogenesis. Not being able to address the concomitant CNS abnormalities in strabismus could be the cause of surgical treatment varying [23] and sometimes unsuccessful results [28, 140–144], often with high recurrence and reoperation rates [145–148], all despite being a common and frequently implemented procedure worldwide [149, 150]. By surgically rearranging EOM position, no steps are taken to enhance control, nor to improve oculomotor and perceptual abilities. If anything, awareness of eye position could be increasingly limited due to the destruction of proprioceptive afferents [151]. Considering that multiple causes, along the periphery-center axis, can be behind the ocular deviation, different treatment strategies tailored to the precise strabismus causes might be needed. Some cases benefiting from surgery, others from patch therapy, glasses prescription, vision therapy, and in some cases, the deviation can resolve spontaneously. Different treatment strategies imply a multidisciplinary approach between ophthalmologists, optometrists, and may be in the future neurologists. Incorporating treatment directed at improving oculomotor control, enhancing fusion, proprioception, interhemispheric connectivity, etc.

## Conclusion

Alterations in structures in the oculomotor and visual systems along the peripheral–central axis can result in strabismus. In concomitant, non-restrictive, developmental strabismus (the most common type of strabismus), no significant anatomical and/or functional abnormalities are present at the level of the EOMs. In these cases, the deviation is believed to be caused by alterations in the central neural pathways involved in visual perception and oculomotor control [152]. However, the exact causes remain poorly understood [20–23]. Multiple arguments reinforce the idea that the SNC plays an important role in comitant strabismus pathogenesis: the higher than average presence of strabismus in neurological conditions and diseases; the increased rates of strabismus in children exposed to neurotoxic substances such as tobacco, alcohol, and drugs during pregnancy; the absence of limitations of gaze; the capacity for some exotropes to converge and some esotropes to diverge; the control and awareness that many strabismic patients have over the deviation; the negative effects stress, fever, and tiredness have in strabismus control; and last but not least, the brain activity changes seen after successful treatment of convergence insufficiency with vision therapy. Furthermore, multiple CNS abnormalities co-exist with strabismus. On the one hand, changes in brain activity, brain connectivity, and gray and white area volumes might be direct consequences of the eye misalignment or its adaptations. On the other hand, these very same functional and anatomical alterations could be the initial cause of strabismus. In order to improve strabismus treatment success rates, research regarding the origins of strabismus should be encouraged so that in the future, treatment is tailored to the precise causes of the eye misalignment. Different treatment methods focusing on distinct points of the peripheral-central axis might allow for a more customized approach and yield better results. With some strabismus cases responding better to periphery-acting treatment (surgery), while other cases showing better outcome with treatment acting at CNS level.
